# A medicare-based comparative mortality analysis of active surveillance in older women with DCIS

**DOI:** 10.1038/s41523-020-00199-0

**Published:** 2020-10-30

**Authors:** Igor Akushevich, Arseniy P. Yashkin, Rachel A. Greenup, E. Shelley Hwang

**Affiliations:** 1 Biodemography of Aging Research Unit, Social Science Research Institute, Durham, NC USA; 2 Department of Surgery, Duke University, Durham, NC USA

**Keywords:** Health care, Epidemiology

## Abstract

Over 97% of individuals diagnosed with ductal carcinoma in situ (DCIS) will choose to receive guideline concordant care (GCC), which was originally designed to treat invasive cancers and is associated with treatment related morbidity. An alternative to GCC is active surveillance (AS) where therapy is delayed until medically necessary. Differences in mortality risk between the two approaches in women age 65+ are analyzed in this study. SEER and Medicare information on treatment during the first year after diagnosis was used to identify three cohorts based on treatment type and timing: GCC (*N* = 21,772; immediate consent for treatment), AS1 (*N* = 431; delayed treatment within 365 days), and AS2 (*N* = 205; no treatment/ongoing AS). A propensity score-based approach provided pseudorandomization between GCC and AS groups and survival was then compared. Strong influence of comorbidities on the treatment received was observed for all age-groups, with the greatest burden observed in the AS2 group. All-cause and breast-cancer-specific mortality hazard ratios (HR) for AS1 were not statistically different from the GCC group; AS2 was associated with notably higher risk for both all-cause (HR:3.54; CI:3.29, 3.82) and breast-cancer-specific (HR:10.73; CI:8.63,13.35) mortality. Cumulative mortality was substantially higher from other causes than from breast cancer, regardless of treatment group. Women managed with AS for DCIS had higher all-cause and breast-cancer-specific mortality. This effect declined after accounting for baseline comorbidities. Delays of up to 12 months in initiation of GCC did not underperform immediate surgery.

## Introduction

The widespread use of mammographic screening in the U.S. has led to an increase in the detection of a previously underdiagnosed condition ductal carcinoma in situ (DCIS). DCIS is considered the earliest detectable form of breast cancer (stage 0). When compared to invasive cancer, it has a lower potential to develop, metastasize, and lead to death. Only 14–53% of diagnosed DCIS cases will progress to invasive cancer^1–3^. However, over 97% of individuals diagnosed with DCIS will choose to receive guideline concordant care (GCC)—a combination of surgery, radiation, and endocrine therapy treatments originally designed to treat invasive cancers. Although receipt of GCC will, in most cases, mitigate the risk of DCIS progression, it is associated with significant treatment-related effects^4^. Moreover, treatment for DCIS has not been shown to reduce overall mortality in large observational studies^5–7^. Thus, surgery may not be the optimal default treatment choice for patients with DCIS whose disease course differs significantly from that of invasive cancer.

Active surveillance (AS) is an alternative to GCC under which no definitive therapy is undertaken at diagnosis. Rather, treatment decisions are based on a watchful waiting paradigm or the observation of the biological behavior of the tumor specific to each individual case. This approach has been adopted as an acceptable standard treatment option for early stage prostate cancer^8–10^ but has not been rigorously evaluated in the case of DCIS. If found to be effective, AS could lead to an improvement in the overall wellbeing of certain categories of DCIS patients by reducing the financial and health burdens associated with preemptive intensive treatment. The use of AS is especially pertinent to patient groups characterized by high levels of comorbidity, such as women age 65+, for whom aggressive treatment may be a suboptimal choice. The purpose of this study was to evaluate the differences in breast-cancer-specific and all-cause mortality between three groups of women age 65+ diagnosed with DCIS: (i) those who initiated GCC within a year of diagnosis and before any evidence of cancer progression was identified; (ii) those who delayed providing consent to GCC treatment until presented with evidence of cancer progression within a year of the initial diagnosis of DCIS; (iii) those who refused GCC treatment for one year or longer. Since the target population is characterized by high levels of comorbidity which may have motivated the lack of treatment, we found it necessary to adjust for patient characteristics that predict treatment choice before examining patient outcomes across the three study groups.

## Results

### Empirical analyses

Frequency distributions for the main characteristics of the patients in GCC, AS1, and AS2 subgroups are presented in Table 1. The hazard ratios of the most important predictors associated with treatment choice presented in Supplementary Table 1. These variables were selected using a backward selection algorithm using a significance level of *p* = 0.15 as the selection criteria for inclusion/exclusion of a factor in groups by year of diagnosis. The variables selected (for at least two time periods) were: (i) three demographic variables (year and age at diagnosis, and marital status), (ii) three cancer diagnosis variables (histology, grade, and ER status), and (iii) five comorbidities (hypertension, myocardial infarction, lung cancer, Alzheimer’s disease/other dementia, and alcohol abuse). The presence of these comorbidities, excepting hypertension was associated with lower likelihood of GCC treatment. Treatment models with and without inclusion of comorbidities were estimated. Prior to pseudorandomization the AS and GCC groups in the original sample were found to be statistically different in 38 out of 62 observed covariates (Supplementary Table 2). After pseudorandomization there were no statistically significant differences in all observed covariates indicating good pseudorandomization quality. The c-indices of the treatment model for the year-of-diagnosis-related time periods (e.g., 1992–1999; 2000–2005; 2006–2011) were 0.803, 0.727, and 0.823 for the model with comorbidities and 0.775, 0.701, and 0.779 for the model without comorbidities (Supplementary Table 3).

**Table 1:** Characteristics of patient groups (%). Area-based variables are shown in Supplementary Table 3.

Variable	GCC	AS1	AS2	Variable	GCC	AS1	AS2
Demography:				Baseline comorbidities:			
Year				Hypertension	81.1	78.6	90.3
1992–1999	27.3	23.3	7.7	MI	3.9	7.0	14.4
2000–2005	40.6	43.5	37.9	Other IHD	33.7	36.7	44.1
2006–2011	32.1	33.3	54.4	Endo/Pericardium	21.2	25.3	37.4
Race				Cardiomyopathy	22.8	25.6	37.9
Black	8.6	8.8	22.6	ARR	35.3	38.6	49.2
Age				HF	15.7	24.0	39.0
65–69	25.5	21.1	10.8	Stroke	23.3	27.0	42.1
70–74	29.5	21.6	15.4	Stroke with complications	4.9	8.6	11.3
75–79	24.0	22.3	20.5	Atherosclerosis	75.6	74.4	79.5
80–84	14.2	21.6	20.5	Peripheral vein	16.9	19.8	23.6
85+	6.8	13.3	32.8	Aneurysm/Embolism/Thrombosis	16.5	21.6	33.3
Geographic area				Non solid caner	2.7	2.8	<5.4
Midwest	17.6	15.8	14.9	Pancreas cancer	0.4	<2.6	<5.4
Northeast	19.7	26.0	14.4	Kidney cancer	0.6	<2.6	<5.4
South	22.2	20.0	22.1	Melanoma	1.2	<2.6	<5.4
West	40.5	38.1	48.7	Lung cancer	2.4	5.3	7.7
Urban vs. Rural				Colorectal cancer	3.1	4.4	5.6
Urban	85.1	80.2	93.8	Other solid slow progressive	14.3	16.3	10.3
Rural	14.9	19.8	6.2	Other solid fast progressive	7.6	9.5	7.2
Marital status				Secondary malignant neoplasm	6.3	10.4	9.7
Married	47.0	32.1	24.1	Other nonspecified Cancers	42.9	42.8	36.9
Other/unknown	53.0	67.9	75.9	COPD	33.2	39.1	44.6
				Pulmonary heart	7.4	9.8	20.5
				Pneumonia	14.4	16.2	27.7
Cancer diagnosis:				Other lung	26.9	33.3	49.7
Histology				Dementia/Alzheimer	7.8	11.6	32.8
8500	63.3	77.4	69.7	Parkinson	1.5	<2.6	<5.4
8501	18.7	8.8	14.9	Depression	16.9	19.8	20.5
8010/8050/8522/850x	18.0	13.7	15.4	Alcohol abuse	1.0	2.6	<5.4
Grade				Drug/Medicine Abuse	0.6	<2.6	<5.4
Well differentiated	12.1	12.1	8.2	Tobacco abuse	11.2	12.1	11.8
Moderately differentiated	24.5	21.4	28.7	Diabetes	30.7	32.8	41.5
Poorly differentiated	21.2	14.0	19.5	Electrolytes	22.0	30.7	44.1
Undifferentiated	9.1	7.2	10.3	Chronic liver disease	9.9	11.2	12.3
Not determined	33.1	45.3	33.3	IBD	10.5	14.0	9.2
ER status				Ulcer	5.8	6.5	7.2
Positive	33.7	20.2	33.4	Gastric bleeding	10.7	12.8	12.8
Negative	8.9	4.7	6.7	Renal disease	15.8	17.4	37.4
Borderline/Unknown	57.3	75.1	59.0	Septicemia	2.4	5.6	9.2
PR status				HIV	0.1	<2.6	<5.4
Positive	27.3	15.6	28.2	Anemia	41.6	45.8	59.5
Negative	13.2	8.4	10.3	Upper/Lower Limb Fracture	53.6	54.2	56.4
Borderline/Unknown	59.5	76.0	61.5	RA	8.5	8.8	8.7
Laterality				Senility	0.4	<2.6	<5.4
Left	51.2	52.3	52.8	Low weight	9.4	12.3	24.1
Right	48.8	47.7	47.2	Obesity	9.9	9.3	13.3

*Since the SEER-Medicare DUA stipulates that the number of individuals less than eleven may not be directly reported or be derivable, we do not report the actual number of individuals and actual frequency for these cells. Instead, the categories “<11” and “2.6%” or “<5.4%” are used.

**The four area-based variables are categorized according their percentiles; therefore each category has 25% of the total sample.

### Treatment effects

**Fig. 1:**
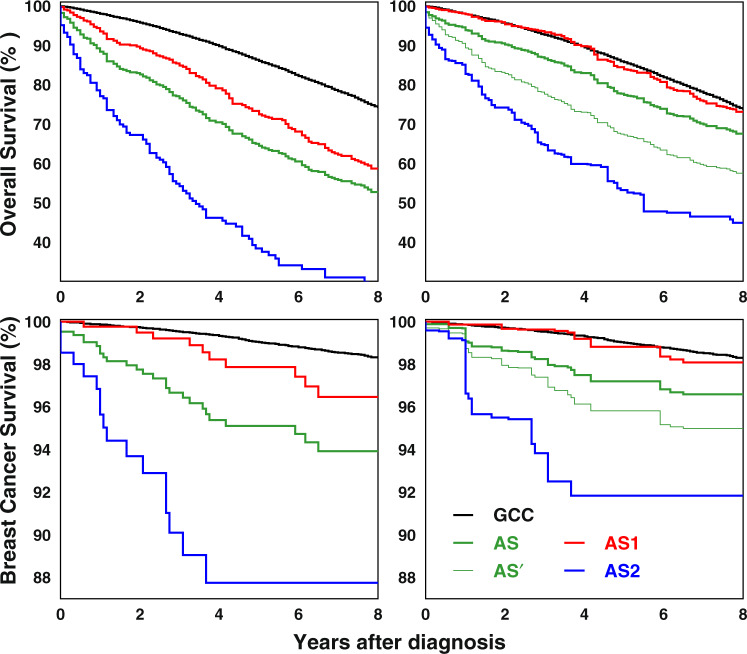
Eight-year DCIS survival for original (left plots) and pseudorandomized (right plots) cohorts (subgroups GCC, AS, AS1, and AS2) created using the propensity-score model. The curve marked by AS’ represents the pseudorandomized cohorts AS with weights calculated without using disease indicators at baseline. CI for the survival curves are shown in Supplementary Fig.1.

In the original sample, AS was found to be associated with significantly higher risk of both all-cause (hazard ratio for 8-year follow-up (HR_8_): 2.39; 95% confidence interval (CI): 2.09–2.72) and breast-cancer mortality (HR_8_: 4.13; CI:2.72–6.28) compared to the GCC group. Survival curves are shown in Fig. 1 and Supplementary Fig. 1. After pseudorandomization, AS was still found to be associated with higher all-cause and breast-cancer mortality risks. Excluding comorbidity from the list of predictors of AS results in higher risk of both overall and breast-cancer-specific mortality.

Analysis of the two AS subgroups showed that group AS2 (untreated) was associated with significant adverse health outcomes, while group AS1 (delayed treatment) was associated with outcomes similar to those observed in the GCC group (Fig. 1). Specifically, there were no statistically significant differences in the overall and BC-specific survival between the GCC and AS1 subgroups (HR:0.99; CI:0.93–1.05 for overall and HR:0.81; CI:0.62–1.05 for breast-cancer-specific survival) in the pseudorandomized populations while group AS2 was associated with significantly increased risk of overall (HR:3.54; CI:3.29–3.82) and BC-specific (HR_8_:10.73; CI:8.63–13.35) mortality.

The results were obtained under two strong assumptions: (i) follow-up starts from the date of diagnosis, and (ii) weights calculated for AS vs. GCC are valid for AS1/AS2. We tested the validity of these assumptions by (i) using a landmark analysis with follow-up starting 1-year after diagnosis (this further reduced the sample sizes to GCC (*N* = 21,425), AS1(*N* = 408), AS2(*N* = 165)), and (ii) generating AS1 vs. GCC and AS2 vs. GCC weights directly (Supplementary Tables 3 and 4). The resulting hazard ratios for these alternative scenarios are presented in Table 2. The results are shown to be stable.

**Table 2:** Overall and breast cancer hazard ratios of being in the group AS, AS1, or AS2 vs. GCC.

Group	Overall survival		Breast cancer survival	
	Original	Weighted	Original	Weighted
Follow-up from the date of diagnosis				
AS	2.39 (2.09, 2.72)	1.31 (1.24, 1.38)	4.13 (2.72, 6.28)	2.15 (1.76, 2.63)
AS1	1.77 (1.49, 2.09)	0.99 (0.93, 1.05)	2.17 (1.15, 4.07)	0.81 (0.62, 1.05)
AS1’		1.17 (1.11, 1.24)		1.04 (0.83, 1.32)
AS2	4.92 (4.01, 6.03)	3.54 (3.29, 3.82)	11.84 (6.90,20.31)	10.73 (8.63,13.35)
AS2’		1.54 (1.46, 1.63)		6.85 (5.76, 8.15)
Follow-up from 1 year after the date of diagnosis				
AS	2.08 (1.79, 2.42)	1.37 (1.29, 1.45)	3.91 (2.48, 6.18)	2.36 (1.91, 2.91)
AS1	1.65 (1.38, 1.99)	1.08 (1.02, 1.15)	2.18 (1.12, 4.24)	0.94 (0.72, 1.23)
AS1’		1.09 (1.03, 1.16)		1.14 (0.90, 1.45)
AS2	3.95 (3.08, 5.05)	3.36 (3.10, 3.63)	11.26 (6.14,20.64)	11.16 (8.89,14.01)
AS2’		1.36 (1.29, 1.45)		3.57 (2.94, 4.35)

“AS1”, “AS2” – estimates are obtained using recalculation of the weights for direct pseudorandomization of AS1 vs. GCC and AS2 vs. GCC.

### Causes of death

**Fig. 2:**
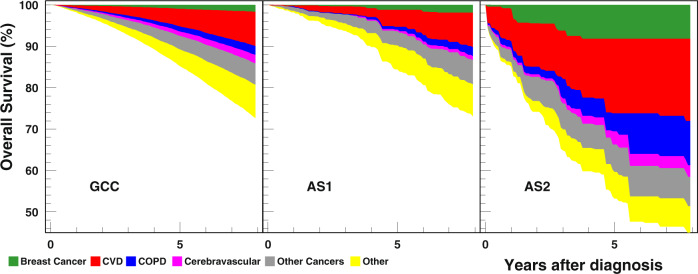
Eight-year cause specific survival among the pseudorandomized group of patients 65+ with diagnosis of DCIS. Panels correspond to Guidelines Concordant Care (right panel), Active Surveillance, Group 1 (center panel), and Active Surveillance, Group 2 (right panel).

The structure of the causes of death for all groups is shown in Fig. 2. The distribution of fractions of 6 causes (breast cancer, CVD, COPD, cerebrovascular diseases, other cancers, and other causes) for patients who did not survive 8 years were (5.9, 29.7, 8.2, 8.0, 21.5, 26.8%) for GCC, (6.7, 29.0, 7.3, 3.6, 23.8, 29.6%) for AS1, and (14.4, 36.6, 16.3, 5.6, 19.7, 7.5%) for AS2. The structure is similar for GCC and AS1, but the fraction of other causes is much smaller in AS2, i.e., more lethal comorbid diseases prevail in its distribution. Eight-year breast cancer survival for pseudorandomized groups were 98.29% (CI:97.98–98.56) for GCC, 98.08% (CI:97.69–98.41) for AS1, and 91.81% (90.54–92.91) for AS2. Mortality significantly increased with age and tumor grade, with lower grade AS2 patients having a breast cancer survival of 91.47% (88.83–93.52) and grade 3 AS2 survival, 84.79% (81.63–87.46) (Supplementary Table 5). The difference between disease specific survival for GCC and AS1 were not statistically significant. One conclusion from Fig. 2 is that cumulative mortality was substantially higher from other causes than from breast cancer, regardless of treatment group.

### Sensitivity study

Alternative models were used to test the stability of our primary analysis including, (i) age group as an additional predictor, (ii) follow-up by age rather than by time after diagnosis, and (iii) follow-up by age with age group as an additional predictor, (iv) an alternative definition of DCIS^11^, (v) using Medicare information to evaluate date of death, and (vi) analyses performed in subgroups defined using demographic factors, place of residence, and comorbidity at baseline. The results are shown in Supplementary Table 5 and demonstrate that the findings of our primary analysis are stable. The finding that there is no statistically significant difference in the overall survival between AS1 and GCC remains valid in the majority of the sensitivity specifications.

## Discussion

In this study, we found that although AS is associated with higher all-cause and breast-cancer mortality much of this extra risk is associated with higher comorbidity levels in the AS population. Unadjusted 5-year overall survival was 86.0% for GCC and only 64.5% for AS. Similarly, unadjusted 5-year breast-cancer-specific survival was 99.03% for GCC and 95.1% for AS. The death hazard ratios significantly increased with age and tumor grade. However, the observed risk decreased dramatically when accounting for baseline comorbidities; after inverse probability weighting, the 5-year breast cancer specific survival improved to 99.00% for GCC and 97.2% for AS. Notably, the 5-year overall survival also differed significantly between GCC (86.0%) and AS (77.4%) groups, supporting that the AS group likely had worse overall clinical status than the GCC group, even after pseudorandomization. This indicates that many unmeasured factors, such as severity of comorbidities, also appropriately contributed to decisions regarding the choice for surgical intervention.

The two subgroups of the AS population which were identified using the additional information derived from Medicare records allowed us to perform a more granular analysis of AS than was possible with SEER data alone. The first group (AS1 – delayed treatment after AS) is characterized by the cessation of AS and initiation of treatment within the first year of diagnosis. Of these, 126 patients (29.2%) had mastectomy with mean time to surgery of 0.813 years; the rest of the sample had lumpectomy with or without radiotherapy. Survival in this group was comparable to that observed in the GCC group for which treatment was administered within 1 year of diagnosis (Fig. 1). This suggests that a delay in initiating treatment does not negatively affect patient survival, therefore the choice of AS as the first course of treatment is unlikely to underperform GCC.

The second subgroup (AS2–no treatment/ongoing AS) continued with AS for at least 1 year after the date of initial diagnosis. This group was characterized by the highest levels of comorbidity and showed significantly worse survival than the AS1 and GCC groups. This group likely includes patients who were not clinically fit for treatment and indeed, at 8-years, only 16% of total mortality in the AS2 group was attributable to breast cancer. The majority of deaths were due to causes other than breast cancer.

There are few manuscripts on clinical outcomes for AS of DCIS. Sanders et al.^12^ found that DCIS, left without treatment, will progress to invasive carcinoma in the same site in the same breast in 30% of patients within 15 years. The Sloan study identified an invasive progression rate of 26% in low/intermediate grade DCIS at a median follow up of 59 months^13^. Stuart et al.^14^ showed that less aggressive treatment leads to higher rates of long-term ipsilateral (in the same breast) local recurrence and breast cancer death rate^13,14^.

Considerations regarding progression as it relates to both overall and disease-specific survival in AS groups are important in the light of discussions on the natural history of the DCIS. It has been estimated that without treatment, between 14 and 53% of DCIS will progress to invasive cancer^1^ over a period of 10 or more years. Mathematical models of the natural history of DCIS indicate that DCIS progression to clinically significant invasive breast cancer is low^2^ and that that AS could be a safe and viable management strategy for carefully selected low risk populations of DCIS patients^3^. These conclusions partly support the findings of Zahl et al.^15^, who raised the possibility that the natural course of some screen-detected invasive breast cancers is to spontaneously regress. Their analysis shows that the incidence in the screened group is higher than in the control in the 6 years follow-up period with the extension to 8 years for sensitivity testing.

We found that cumulative mortality in the older patient population with DCIS is substantially higher from other causes than from breast cancer, regardless of treatment group. The ratio of other cause to breast cancer mortality is 12.90 for GCC and 6.13 for AS. This ratio increases with age up to 85. We detected no significant differences in this ratio between original and pseudorandomized cohorts. The age patterns for these ratios were in agreement with those found in Ryser et al.^3^. The results of subgroup analyses for age and grade-specific groups were expected and generally confirm findings of a prior study by Sagara et al.^16^ which reported a significantly lower breast cancer specific survival benefit with surgery for low-grade DCIS, compared to intermediate- or high-grade DCIS.

Age and comorbidity mix strongly influence whether AS is the first course of treatment. Fractions of AS shown in Table 2 were approximately uniformly distributed among age groups (about 1/5 for each of five groups used) while the fractions of GCC rapidly dropped starting from age 75. The OR of GSS vs. AS were about 3.0 for ages 65–80 vs age 85+. Almost all comorbidities increased the probability of having AS. Comorbid diseases with high mortality had higher differences between frequencies of AS and GCC. These results are consistent with prior research: Kimmick et al.^17^ showed that greater comorbidity burden is associated with a lower rate of GCC, and Schonberg et al.^18^ demonstrated that treatment choices were significantly associated with age and comorbidity, with age as the stronger predictor. Other factors that influenced treatment course were having a tumor grade of “not differentiated,” living in an area with a low proportion of African Americans, and living in a SEER region on the West coast of the U.S. These findings are similarly in agreement with previous work^19^.

Although this study reports on one of the largest cohorts of AS for DCIS, the sample size precluded a comprehensive evaluation of tumor characteristics and outcome. In other studies, nuclear grade (high, intermediate, or low), necrosis (presence or absence), and polarization (architectural differentiation) were named important prognostic features for the progression of DCIS into invasive cancer^12,20^. Lopez-Garcia et al.^21^ reviewed the available molecular data on breast cancer risk indicator and precursor lesions, the putative mechanisms of progression from in situ to invasive disease. They concluded that the molecular data available on in situ lesions suggest that they are at least as heterogeneous as their invasive counterparts and proposed a revised model of breast cancer evolution. In our study cohort, we found that in the AS2 group, increased DSS was associated with higher grade, supporting the role of early intervention in this group. However, we note that the potential benefit of surgery in this study cohort may be overestimated compared to the overall DCIS population, as a greater proportion of patients 70 and older are diagnosed with DCIS on the basis of symptoms, rather than on screening.

Additional research is necessary to be able to fully address the full range of secondary questions that a patient may have after receiving a DCIS diagnosis and choosing between AS and GCC. The most immediately obvious concerns may include: (i) how does the choice of GCC/AS impact the risk of developing invasive cancer? (ii) what are the likely effects of GCC/AS on quality-of-life and financial well-being? (iii) how do specific comorbidities as well as other risks or protective factors modify the expected outcomes of GCC/AS? and (iv) how should these benefits and risks of treatment for DCIS be considered in terms of the risk of treatment-related comorbidities and future overall health? Analyses of these and other questions require a more expanded range of data than what is available in cancer registries, especially SEER, which only provides information on the first course of therapy. For example, the identification of invasion in a patient originally diagnosed with DCIS who has been undergoing AS for 2 years requires an algorithmic approach to Medicare analysis because it is not reported in SEER and cannot be directly observed in SEER-Medicare. These limitations may be addressable with data from ongoing studies aimed at determining whether some DCIS patients with fewer comorbidities may safely opt for AS^22^.

Due to its retrospective nature and reliance on administrative data this study has a number of important limitations. Although this study is generalizable to the 65+ population, all patients in this study had coverage via Medicare, and therefore the results are not necessarily generalizable to population of patients that may include younger, uninsured, or underinsured patients. However, use of the population-based SEER–Medicare database has the significant advantage of allowing the evaluation of a large number of patients in a specific disease stage subset. It is not likely that a prospective study that includes a similar number of elderly patients with this specific tumor stage could ever be performed. The length of the “AS” in the AS groups was limited to 1 year, therefore we are not able to differentiate by time to treatment and/or lack of treatment after this period and do not monitor for the occurrence of surveillance-related procedures. This was dictated by the need to harmonize the two data sources used in this study – SEER and Medicare – and can be improved upon in a future Medicare-only study. Although a wide diversity of clinically interesting endpoints other than death exist, we were limited to the information available in the data and consistent with our study design involving harmonization of SEER and Medicare measures. A future LORIS^23^ and/or COMET^24^-based study may be able to overcome this limitation and include a wider range of endpoints, but this is not feasible under the current study design. Finally, we do not include endocrine therapy in our definition of receiving “treatment” and, therefore, it was not possible to determine the impact of systemic treatment in this cohort.

In conclusion, our study provides new knowledge on the effect of comorbidities on treatment selection at time of DCIS diagnosis as well as the combined effect of the comorbidity mix and treatment choice on subsequent patient survival. We found that after accounting for differences in comorbidity at time of diagnosis, much of the differences in mortality risk associated with the choice of AS vs. GCC are mitigated. Further, when treatment is initiated within 1 year of diagnosis (AS1 treatment group) the all-cause and breast cancer mortality rates do not differ from the GCC group. In contrast, those who continue with AS past the 1-year demonstrate increased risk of both all-cause and BC caused mortality. Our findings support that recommendations for DCIS treatment in an older patient population should acknowledge that the burden of comorbidities, have a far greater impact on overall survival than DCIS treatment. Therefore, thoughtfully accounting for risk profiles in elderly individuals diagnosed with DCIS will allow for progress in reducing the burden of this diagnosis on patients, while minimizing exposure to undue risks.

## Methods

### Data

Data drawn from the Surveillance, Epidemiology, and End Results program linked to administrative health insurance claims records from the Medicare program (SEER-Medicare) was used for this study. The SEER-Medicare population is approximately representative of the U.S. but demonstrates somewhat higher proportions of minorities, urban residents, and higher socio-economic status^25^. SEER-Medicare provides data on the date of diagnosis, histology, stage, and grade of the tumor as well as the therapy recommended and/or provided within 1 year of diagnosis, follow-up vital status, cause of death (if applicable), and basic demographic and area-based socio-economic characteristics. The Medicare component provides additional information on the diagnoses made (International Classification of Disease 9th Edition, Clinical Modification (ICD-9)) and procedures performed (Procedural ICD-9, Current Procedural Terminology 4th Edition (CPT-4) and Healthcare Common Procedure Coding System) on all episodes of care paid for by Medicare Parts A and B on a fee-for-service basis.

### Sample selection

To ensure Medicare coverage, we restricted the sample pool to females who were age 65+ at the time of their breast cancer diagnosis over the 1992–2011 period (*N* = 381,056). Of these, 36,202 (9.50%) were diagnosed with DCIS (histology codes 8500–8509, 8010, 8050, or 8522 and nonmalignant tumor behavior). To address concerns of under-identification of treatment in Medicare data due to lack of insurance coverage and possible discrepancies between diagnosis dates found in Medicare data and the SEER registry (likely caused by billing lags), we required each DCIS patient to have: an administrative claim for breast cancer (ICD-9: 174 or 233.0) 12 months before or after the SEER diagnosis date (8,761 excluded), coverage under the traditional Medicare fee-for-service system (2327 excluded) and continuous Medicare Part A and B coverage (2485 excluded) in the period covering 12 months before and after the SEER registry date of DCIS diagnosis (unless the reason for lack of coverage was death after diagnosis). Sequential application of each restriction resulted in 27,441, 25,114, and 22,629 patients. Finally, we excluded 53 patients with missing data on treatment choice in SEER. The final sample contained 22,576 female patients diagnosed with DCIS (Supplementary Fig. 2).

### Treatment measures

**Fig. 3:**
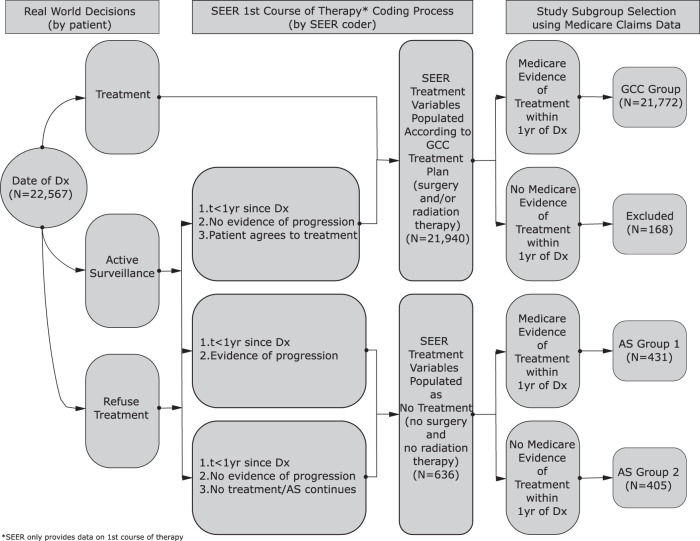
Patient selection and treatment group definitions.

Using information from the SEER on the first course of treatment (the only information on cancer treatment provided by SEER) and claims-based information on treatment derived from Medicare records (Supplementary Table 6) we developed an algorithm to categorize our sample into four mutually exclusive treatment groups (Fig. 3). The first group, GCC, included individuals who agreed to undergo GCC within 1 year of diagnosis conditional on no evidence of cancer progression being identified between the initial diagnosis date and treatment initiation. The second group, AS1, included individuals who agreed to undergo treatment within 1 year of diagnosis but only initiated treatment after being presented with evidence of cancer progression. This group can be called the “true AS” group as based on the way SEER data is generated we can be certain that (i) treatment was refused at time of diagnosis and (ii) surveillance did in fact occur as treatment was initiated only after evidence of cancer progression was obtained. Group AS1 is also characterized by delay of treatment by a period of no longer than 365 days and can be treated as a “delayed treatment” group for the purposes of interpretation. Group 3, AS2, included individuals who refused (or were not recommended) treatment at time of diagnosis and did not initiate treatment within 1 year of diagnosis. Information on cancer progression and/or the presence of screening procedures to support ongoing systematic surveillance was not identified for this group. Group 4, Excluded, contained individuals from the SEER database for whom no accompanying Medicare records could be identified. The use of a 1-year period for group assignment was necessitated by our need to harmonize the two sources of information – SEER and Medicare – used in our study. The treatment choice entry in the SEER database (most importantly the “no treatment” entry) is determined by both patient behavior (agree/disagree to undergo treatment), a fixed time frame (365 from time of diagnosis) and the behavior of the tumor (progression prior to treatment initiation or not). Therefore, when we used Medicare records to refine the information drawn from SEER into more granular and informative categories, the same time frame had to be used. The group-specific initial sample sizes were: GCC (*N* = 21,772), AS (*N* = 636) of which AS1 (*N* = 431) and AS2 (*N* = 205). The Excluded group contained a further 168 entries. The logic used in sample selection is demonstrated in Fig. 3. Finally, we excluded individuals who died in the same month as their cancer diagnosis (e.g., 0 month follow-up). This reduced the sample sizes to: GCC (*N* = 21,762), AS1 (*N* = 430), AS2 (*N* = 195).

### Statistical analysis

In order to make GCC and AS groups comparable with each other and to account for non-treatment-related differences, we applied a pseudorandomization algorithm^26–28^. To account for the maximum possible number of sources of heterogeneity we used a comprehensive set of demographic, cancer diagnosis, socio-economic, and comorbidity-related characteristics (Supplementary Table 7^29^). A logistic model was then used to predict the specific type of cancer treatment (i.e., AS vs. GCC) for each patient. The resulting pseudorandomized subgroups were well matched on all variables (Supplementary Table 2). Based on the results of this model, individual weights (also known as inverse-probability weights, IPW) were then calculated as the reciprocal of the probability to have actually observed GCC or AS treatment resulting in a weighted population pseudorandomized with respect to all predictors used in the treatment model. Use of IPW ensures that the AS and GCC groups identified in our sample contain no statistically significant differences in terms of the variables included in the treatment model. Supplementary Table 2 provides summary statistics as well as significance and pseudorandomization quality testing for all 62 variables involved in pseudorandomizing the AS and GCC groups. After pseudorandomization, the only observed differences in mortality would be due to the choice of treatment (AS vs GCC) or some factors not measured or collected in the study data. Finally, the causal effect of the treatment modes was evaluated using the Cox proportional hazards model. The only explanatory variable included was treatment group, as all other observable covariates were controlled for in the pseudorandomization process. Survival time was defined by the respective SEER variable and the cause-of-death code was used to determine breast-cancer death cases.

We note that pseudorandomization of GCC and AS does not guarantee that any given subset of AS (e.g., AS1, AS2) is well matched to GCC. Therefore, we performed an additional sensitivity analysis by regenerating IPW for GCC vs. AS1 and GCC vs. AS2 directly. Supplementary Tables 3 and 4 provides the respective pre/post pseudorandomization group characteristics and tests of IPW quality.

The Duke University Institutional Review Board approved the protocol used in this study. All analyses were done using SAS 9.4 software, copyright SAS Institute Inc.

### Reporting summary

Further information on research design is available in the Nature Research Reporting Summary linked to this article.

## Supplementary information

Supplementary Information

Reporting Summary

## Data Availability

The data generated and analyzed during this study are described in the following metadata record: 10.6084/m9.figshare.12776825^30^. All the datasets analyzed in the current study are in SAS file format. SEER-MEDICARE data were used to generate all the tables and figures in this article. The Centers for Medicare and Medicaid Services do not allow the redistribution of their data by researchers. SEER-MEDICARE data are distinct from the publicly available SEER database, and can be obtained by researchers, by following the process described on https://healthcaredelivery.cancer.gov/seermedicare/obtain/requests.html (access requirements include Institutional Review Board approval, and the completion of a Data Use Agreement). Please note that the process and range of data availability has changed in 2020 and therefore obtaining some of earlier years of data used in this study may prove a challenge.

## Code availability

The Centers for Medicare and Medicaid Services do not allow the redistribution of their data by researchers. However, upon request and after confirmation that the requestor has the appropriate Research Identifiable (RIF) Data Use Agreement (DUA) in place, as well as IRB approval at his/her institution, the authors will provide SAS code that will allow the re-generation of the data in a form appropriate to the replication of the current analysis.
