# Supplementary material for: A medicare-based comparative mortality analysis of active surveillance in older women with DCIS
Source: NPJ Breast Cancer. 2020 Oct 30;6:57. doi: 10.1038/s41523-020-00199-0 (PMC7599206; doi:10.1038/s41523-020-00199-0)
Supplement: Supplementary file 1 — Supplementary Information [file 41523_2020_199_MOESM1_ESM.pdf]

## Supplementary Material

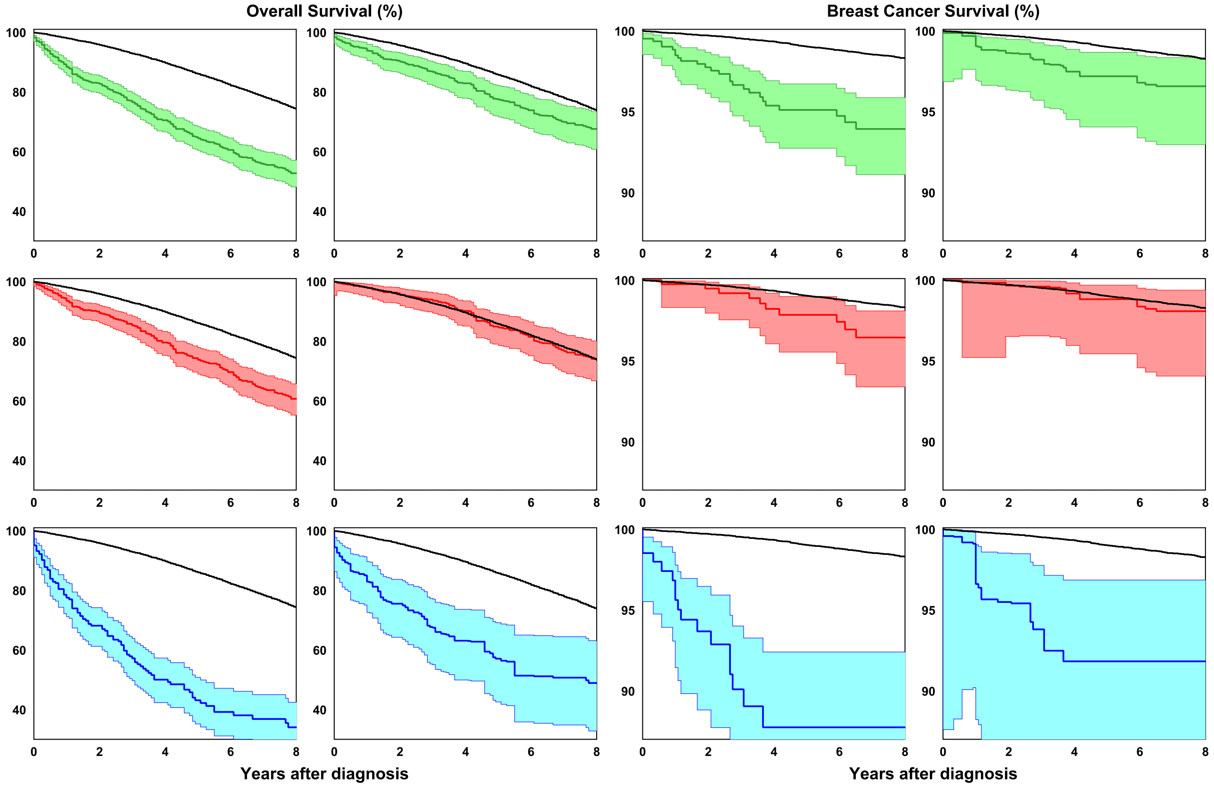


**Supplementary Figure 1:** Eight-Year DCIS Survival for original and pseudorandomized cohorts (subgroups AS, AS1, and AS2 from the top to bottom vs. GCC) with confidence intervals

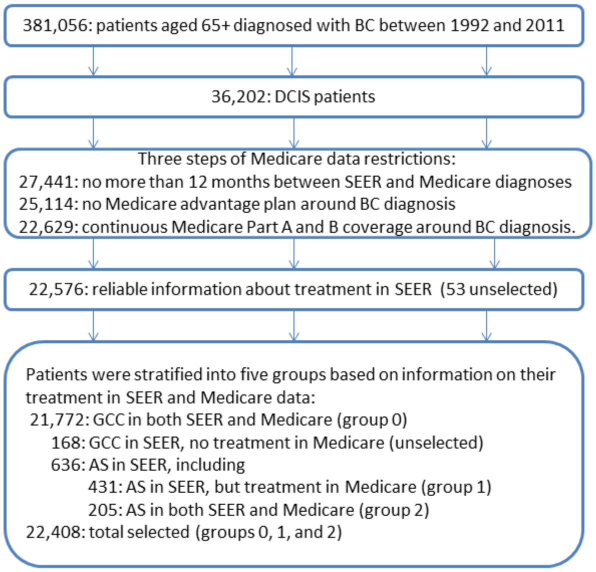


**Supplementary Figure 2:** Patient selection and treatment group definitions

**Supplementary Table 1**. GCC hazard ratios for the year group 2006-2011

| Category | Including diseases OR | Including diseases p-value | Excluding diseases OR | Excluding diseases p-value |
|---|---|---|---|---|
| Year of diagnosis | 0.84 | <.0001 | 0.85 | <.0001 |
| White vs. non-White | 1.98 | 0.0008 | 2.06 | 0.0002 |
| Age: 65-69 vs. 85+ | 3.70 | <.0001 | 3.78 | <.0001 |
| Age: 70-74 vs. 85+ | 3.39 | <.0001 | 3.52 | <.0001 |
| Age: 75-79 vs. 85+ | 3.11 | <.0001 | 3.17 | <.0001 |
| Age: 80-84 vs. 85+ | 2.40 | <.0001 | 2.41 | <.0001 |
| Midwest vs. West | 0.98 | 0.9214 | 0.93 | 0.7221 |
| Northeast vs. West | 1.13 | 0.4938 | 1.20 | 0.3179 |
| South vs. West | 2.06 | 0.0002 | 1.98 | 0.0002 |
| Married vs. Unmarried or Unknown | 1.79 | 0.0002 | 1.83 | <.0001 |
| Histology: 8010 vs. 8522 | 0.09 | 0.0122 | 0.09 | 0.0119 |
| Histology: 8050 vs. 8522 | 0.21 | 0.1347 | 0.22 | 0.1349 |
| Histology: 8500 vs. 8522 | 0.11 | 0.0021 | 0.12 | 0.0028 |
| Histology: 8501 vs. 8522 | 0.25 | 0.0723 | 0.28 | 0.0938 |
| Histology: 850* vs. 8522 | 0.21 | 0.0395 | 0.21 | 0.0380 |
| Grade: differentiate vs. not determined | 1.79 | 0.0115 | 1.74 | 0.0138 |
| Grade: moderately differentiated vs. not determined | 1.56 | 0.0172 | 1.59 | 0.0102 |
| Grade: poorly differentiated vs. not determined | 1.81 | 0.0041 | 1.69 | 0.0089 |
| Grade: undifferentiated vs. not determined | 2.22 | 0.0099 | 2.09 | 0.0147 |
| ERA: Positive vs. Unknown | 3.50 | <.0001 | 3.35 | <.0001 |
| ERA: Negative vs. Unknown | 5.10 | <.0001 | 4.69 | <.0001 |
| Hypertension | 1.59 | 0.0263 | | |
| MI | 0.53 | 0.0171 | | |
| Other IHD | 1.33 | 0.0747 | | |
| Stroke with complications | 0.69 | 0.1126 | | |
| Aneurysm/Arterial Embolism/Thrombosis | 0.78 | 0.1227 | | |
| Lung Cancer | 0.29 | <.0001 | | |
| Secondary malignant neoplasm | 0.53 | 0.0118 | | |
| Other Non-specified Cancer | 1.42 | 0.0133 | | |
| Pulmonary Heart | 0.59 | 0.0093 | | |
| Dementia/Alzheimer | 0.52 | 0.0004 | | |
| Alcohol abuse | 0.33 | 0.0062 | | |
| HIV | 0.04 | <.0001 | | |
| RA | 2.08 | 0.0181 | | |
| Area based: Lower-poverty 1 vs. 4 (in percentiles) | 1.83 | 0.0486 | 1.73 | 0.0682 |
| Area based: Lower-poverty 2 vs. 4 | 1.80 | 0.0557 | 1.79 | 0.0575 |
| Area based: Lower-poverty 3 vs. 4 | 2.02 | 0.0318 | 1.91 | 0.0456 |
| Area based: Lower-fraction-of-blacks 1 vs. 4 | 1.83 | 0.0486 | 1.73 | 0.0682 |
| Area based: Lower-fraction-of-blacks 2 vs. 4 | 1.80 | 0.0557 | 1.79 | 0.0575 |
| Area based: Lower-fraction-of-blacks 3 vs. 4 | 2.02 | 0.0318 | 1.91 | 0.0456 |
| Area based: More educated 1 vs. 4 | 1.45 | 0.0629 | 1.47 | 0.0541 |
| Area based: More educated 2 vs. 4 | 1.22 | 0.3044 | 1.25 | 0.2356 |
| Area based: More educated 3 vs. 4 | 0.94 | 0.7189 | 0.94 | 0.7401 |

**Supplementary Table 2:** Characteristics of original cohort (ORC) and pseudorandomized cohorts (PSC) for GCC and AS patients

| Variable | Frequency | | Percent | | | | p-value | |
|---|---|---|---|---|---|---|---|---|
| | ORC or PSC | | ORC | | PSC | | ORC | PSC |
| | GCC | AS | GCC | AS | GCC | AS | | |
| **Demographic Variables** | | | | | | | | |
| **Year of cancer diagnosis:** 1992-1999 | 5943 | 115 | 27.31 | 18.4 | 27.06 | 29.67 | <.0001 | 0.6750 |
| 2000-2005 | 8838 | 261 | 40.61 | 41.76 | 40.63 | 40.68 | | |
| 2006-2011 | 6981 | 249 | 32.08 | 39.84 | 32.31 | 29.65 | | |
| **Black** | 1868 | 82 | 8.58 | 13.12 | 8.7 | 13.46 | <.0001 | 0.0829 |
| **Age at diagnosis**: 65-69 | 5547 | 112 | 25.49 | 17.92 | 25.29 | 30.16 | <.0001 | 0.4369 |
| 70-74 | 6425 | 123 | 29.52 | 19.68 | 29.24 | 26.82 | | |
| 75-79 | 5222 | 136 | 24 | 21.76 | 23.93 | 21.63 | | |
| 80-84 | 3094 | 133 | 14.22 | 21.28 | 14.41 | 13.12 | | |
| 85+ | 1474 | 121 | 6.77 | 19.36 | 7.13 | 8.28 | | |
| **Geographic Area**: Midwest | 3831 | 97 | 17.6 | 15.52 | 17.56 | 20.73 | 0.1985 | 0.5430 |
| Northeast | 4277 | 140 | 19.65 | 22.4 | 19.72 | 21.88 | | |
| South | 4840 | 129 | 22.24 | 20.64 | 22.22 | 19.87 | | |
| West | 8814 | 259 | 40.5 | 41.44 | 40.5 | 37.52 | | |
| **Urban vs. Rural**: Metropolitan | 18524 | 528 | 85.12 | 84.48 | 85.09 | 84.59 | 0.6573 | 0.8438 |
| Nonmetropolitan | 3238 | 97 | 14.88 | 15.52 | 14.91 | 15.41 | | |
| **Marital Status**: Married | 10227 | 185 | 46.99 | 29.6 | 46.51 | 49.29 | <.0001 | 0.4526 |
| Other/ Unknown marital status | 11535 | 440 | 53.01 | 70.4 | 53.49 | 50.71 | | |
| **Cancer Diagnosis characteristics** | | | | | | | | |
| **Histology**: 8500 | 13769 | 469 | 63.27 | 75.04 | 63.61 | 69.57 | <.0001 | 0.2832 |
| 8501 | 4071 | 67 | 18.71 | 10.72 | 18.48 | 15.69 | | |
| 8010/8050/8522/850x | 3922 | 89 | 18.02 | 14.24 | 17.91 | 14.74 | | |
| **Grade**: differentiate | 2623 | 68 | 12.05 | 10.88 | 12.03 | 14.24 | <.0001 | 0.7316 |
| moderately differentiated | 5336 | 148 | 24.52 | 23.68 | 24.5 | 22.22 | | |
| poorly differentiated | 4617 | 98 | 21.22 | 15.68 | 21.05 | 21.44 | | |
| undifferentiated | 1985 | 51 | 9.12 | 8.16 | 9.1 | 7.28 | | |
| not determined | 7201 | 260 | 33.09 | 41.6 | 33.32 | 34.83 | | |
| **ER Status**: Positive | 7344 | 154 | 33.75 | 24.64 | 33.48 | 28.53 | <.0001 | 0.3976 |
| Negative | 1941 | 33 | 8.92 | 5.28 | 8.81 | 10.77 | | |
| Borderline /Unknown | 12477 | 438 | 57.33 | 70.08 | 57.71 | 60.7 | | |
| **PR status**: Positive | 5947 | 122 | 27.33 | 19.52 | 27.1 | 21.09 | <.0001 | 0.1137 |
| Negative | 2872 | 56 | 13.2 | 8.96 | 13.06 | 17.15 | | |
| Borderline/Unknown | 12943 | 447 | 59.48 | 71.52 | 59.84 | 61.76 | | |
| **Laterality**: Left | 11143 | 328 | 51.2 | 52.48 | 51.19 | 53.17 | 0.5292 | 0.5966 |
| Right | 10619 | 297 | 48.8 | 47.52 | 48.81 | 46.83 | | |
| **Baseline Comorbidities** | | | | | | | | |
| Hypertension | 17656 | 514 | 81.13 | 82.24 | 81.19 | 81.67 | 0.4850 | 0.8660 |
| MI | 842 | 58 | 3.87 | 9.28 | 4.02 | 3.43 | <.0001 | 0.4389 |
| Other IHD | 7329 | 244 | 33.68 | 39.04 | 33.88 | 32.23 | 0.0052 | 0.6409 |
| Endo/Pericardium | 4613 | 182 | 21.2 | 29.12 | 21.39 | 20.17 | <.0001 | 0.6471 |
| Cardiomyopathy | 4952 | 184 | 22.76 | 29.44 | 22.99 | 22.26 | <.0001 | 0.8102 |
| ARR | 7672 | 262 | 35.25 | 41.92 | 35.48 | 33.88 | 0.0006 | 0.6525 |
| HF | 3425 | 179 | 15.74 | 28.64 | 16.05 | 18.17 | <.0001 | 0.3884 |
| Stroke | 5076 | 198 | 23.33 | 31.68 | 23.58 | 20.78 | <.0001 | 0.2669 |
| Stroke with complications | 1062 | 59 | 4.88 | 9.44 | 4.98 | 5.01 | <.0001 | 0.9760 |
| Atherosclerosis | 16442 | 475 | 75.55 | 76 | 75.61 | 77.36 | 0.7980 | 0.5737 |
| Peripheral Vein | 3679 | 131 | 16.91 | 20.96 | 17.01 | 15.68 | 0.0078 | 0.5113 |
| Aneurysm/Arterial Embolism/Thrombosis | 3592 | 158 | 16.51 | 25.28 | 16.7 | 18.59 | <.0001 | 0.4803 |
| Non-Solid Caner | 588 | 19 | 2.7 | 3.04 | 2.71 | 2.41 | 0.6079 | 0.7041 |
| Pancreas Cancer | 85 | <11 | 0.39 | <1.8 | 0.41 | <1.8 | 0.0274 | 0.5904 |

| | | | | | | | | |
|---|---|---|---|---|---|---|---|---|
| Kidney Cancer | 141 | <11 | 0.65 | <1.8 | 0.65 | <1.8 | 0.9806 | 0.0905 |
| Melanoma | 269 | <11 | 1.24 | <1.8 | 1.24 | <1.8 | 0.7954 | 0.5013 |
| Lung Cancer | 523 | 38 | 2.4 | 6.08 | 2.49 | 2.49 | <.0001 | 0.9983 |
| Colorectal Cancer | 685 | 30 | 3.15 | 4.8 | 3.17 | 3.36 | 0.0206 | 0.8324 |
| Other Solid Slow Progressive | 3112 | 90 | 14.3 | 14.4 | 14.32 | 13.34 | 0.9440 | 0.7119 |
| Other Solid Fast Progressive | 1662 | 55 | 7.64 | 8.8 | 7.68 | 5.58 | 0.2815 | 0.1571 |
| Secondary malignant neoplasm | 1378 | 64 | 6.33 | 10.24 | 6.4 | 7.12 | <.0001 | 0.6082 |
| Other Non-specified Cancer | 9334 | 256 | 42.89 | 40.96 | 42.86 | 41.85 | 0.3361 | 0.7878 |
| COPD | 7219 | 255 | 33.17 | 40.8 | 33.36 | 37.08 | <.0001 | 0.2871 |
| Pulmonary Heart | 1609 | 82 | 7.39 | 13.12 | 7.58 | 6.5 | <.0001 | 0.3546 |
| Pneumonia | 3123 | 124 | 14.35 | 19.84 | 14.52 | 12.69 | 0.0001 | 0.3810 |
| Other Lung | 5845 | 240 | 26.86 | 38.4 | 27.17 | 27.2 | <.0001 | 0.9921 |
| Dementia/Alzheimer | 1701 | 114 | 7.82 | 18.24 | 8.12 | 8.45 | <.0001 | 0.8030 |
| Parkinson | 329 | 18 | 1.51 | 2.88 | 1.53 | 1.37 | 0.0063 | 0.7348 |
| Depression | 3688 | 125 | 16.95 | 20 | 17.08 | 12.72 | 0.0453 | 0.0196 |
| Alcohol Abuse | 220 | 14 | 1.01 | 2.24 | 1.05 | 0.76 | 0.0029 | 0.4437 |
| Drug/Medicine Abuse | 122 | <11 | 0.56 | <1.8 | 0.58 | <1.8 | 0.7937 | 0.3192 |
| Tobacco Abuse | 2448 | 75 | 11.25 | 12 | 11.3 | 9.31 | 0.5583 | 0.2763 |
| Diabetes | 6674 | 222 | 30.67 | 35.52 | 30.77 | 28.54 | 0.0096 | 0.4660 |
| Electrolytes | 4779 | 218 | 21.96 | 34.88 | 22.28 | 22.38 | <.0001 | 0.9682 |
| Chronic Liver Disease | 2144 | 72 | 9.85 | 11.52 | 9.87 | 11.58 | 0.1686 | 0.4700 |
| IBD | 2280 | 78 | 10.48 | 12.48 | 10.57 | 8.74 | 0.1078 | 0.2339 |
| Ulcer | 1258 | 42 | 5.78 | 6.72 | 5.82 | 6.89 | 0.3222 | 0.5851 |
| Gastric Bleeding | 2335 | 80 | 10.73 | 12.8 | 10.81 | 10.69 | 0.1000 | 0.9508 |
| Renal Disease | 3428 | 148 | 15.75 | 23.68 | 15.98 | 16.19 | <.0001 | 0.9381 |
| Septicemia | 529 | 42 | 2.43 | 6.72 | 2.55 | 2.24 | <.0001 | 0.5994 |
| HIV | 15 | <11 | 0.07 | <1.8 | 0.09 | <1.8 | <.0001 | 0.7289 |
| Anemia | 9046 | 313 | 41.57 | 50.08 | 41.83 | 38.03 | <.0001 | 0.2631 |
| Upper/Lower Limb Fracture | 11669 | 343 | 53.62 | 54.88 | 53.74 | 46.96 | 0.5337 | 0.0604 |
| RA | 1848 | 55 | 8.49 | 8.8 | 8.49 | 10.34 | 0.7854 | 0.5056 |
| Senility | 90 | <11 | 0.41 | <1.8 | 0.42 | <1.8 | 0.1429 | 0.6112 |
| Low Weight | 2041 | 100 | 9.38 | 16 | 9.57 | 10.8 | <.0001 | 0.5958 |
| Obesity | 2148 | 66 | 9.87 | 10.56 | 9.87 | 11.95 | 0.5691 | 0.4303 |
| **Area-based measures:** whose living area is characterized by … | | | | | | | | |
| **Lower-poverty-level**: 1st percentile | 5446 | 155 | 25.03 | 24.8 | 25.03 | 26.2 | 0.6497 | 0.5595 |
| 2nd percentile | 5453 | 146 | 25.06 | 23.36 | 25.02 | 28.41 | | |
| 3rd percentile | 5439 | 156 | 24.99 | 24.96 | 25 | 21.38 | | |
| 4th percentile | 5424 | 168 | 24.92 | 26.88 | 24.94 | 24.01 | | |
| **Higher income**: 1st percentile | 5444 | 159 | 25.02 | 25.44 | 25.05 | 26.84 | 0.2454 | 0.8004 |
| 2nd percentile | 5466 | 138 | 25.12 | 22.08 | 25.1 | 22.45 | | |
| 3rd percentile | 5431 | 155 | 24.96 | 24.8 | 24.94 | 26.52 | | |
| 4th percentile | 5421 | 173 | 24.91 | 27.68 | 24.91 | 24.19 | | |
| **Lower-fraction-of-black population**: 1st percentile | 5527 | 134 | 25.4 | 21.44 | 25.31 | 27.99 | 0.0001 | 0.6384 |
| 2nd percentile | 5427 | 127 | 24.94 | 20.32 | 24.83 | 21.43 | | |
| 3rd percentile | 5416 | 167 | 24.89 | 26.72 | 24.96 | 24.07 | | |
| 4th percentile | 5392 | 197 | 24.78 | 31.52 | 24.9 | 26.52 | | |
| **More educated**: 1st percentile | 5456 | 147 | 25.07 | 23.52 | 25.11 | 27.24 | 0.4655 | 0.8690 |
| 2nd percentile | 5434 | 165 | 24.97 | 26.4 | 24.97 | 23.85 | | |
| 3rd percentile | 5423 | 167 | 24.92 | 26.72 | 24.93 | 23.12 | | |
| 4th percentile | 5449 | 146 | 25.04 | 23.36 | 24.99 | 25.79 | | |

*Since The SEER-Medicare DUA stipulates that the number of individuals less than eleven may not be directly reported or be derivable, we do not report the actual number of individuals and actual frequency for these cells. Instead the categories “<11” and “<1.8%” are used.

**Supplementary Table 3**. C-indices of treatment modes

| Starting follow-up time | Model | c-index | | | | | |
|---|---|---|---|---|---|---|---|
| | | With comorbidities | | | Without comorbidities | | |
| | | 1992-1999 | 2000-2005 | 2006-2011 | 1992-1999 | 2000-2005 | 2006-2011 |
| One month | AS vs. GSS | 0.803 | 0.727 | 0.823 | 0.775 | 0.701 | 0.779 |
| One month | AS1 vs. GSS | 0.807 | 0.716 | 0.806 | 0.774 | 0.696 | 0.786 |
| One month | AS2 vs. GSS | 0.948 | 0.881 | 0.855 | 0.826 | 0.832 | 0.823 |
| One year | AS vs. GSS | 0.800 | 0.708 | 0.818 | 0.764 | 0.698 | 0.783 |
| One year | AS1 vs. GSS | 0.803 | 0.708 | 0.806 | 0.770 | 0.691 | 0.801 |
| One year | AS2 vs. GSS | 0928 | 0.866 | 0.848 | 0.836 | 0.824 | 0.819 |

**Supplementary Table 4.** P-values of comparison of characteristics of original cohort (ORC) and pseudorandomized cohorts (PSC) for GCC and AS, AS1, or AS2 patients for initial follow-up 1 month and 1 year

| Variable | 1mon, AS1 | | 1mon, AS2 | | 1year, AS | | 1year, AS1 | | 1year, AS2 | |
|---|---|---|---|---|---|---|---|---|---|---|
| | ORC | PSC | ORC | PSC | ORC | PSC | ORC | PSC | ORC | PSC |
| Year of Cancer Diagnosis | 0.1667 | 0.9842 | <.0001 | 0.8652 | <.0001 | 0.8578 | 0.2036 | 0.8722 | <.0001 | 0.8877 |
| Black | 0.8527 | 0.7906 | <.0001 | 0.0265 | 0.0004 | 0.9093 | 0.8162 | 0.7232 | <.0001 | 0.2521 |
| Age at Diagnosis | <.0001 | 0.0782 | <.0001 | 0.8738 | <.0001 | 0.2703 | <.0001 | 0.1003 | <.0001 | 0.5774 |
| Geographic Area | 0.0116 | 0.7532 | 0.0785 | 0.1100 | 0.1791 | 0.6177 | 0.0110 | 0.5209 | 0.1017 | 0.2905 |
| Urban vs. Rural | 0.0049 | 0.2143 | 0.0006 | 0.0219 | 0.2105 | 0.8207 | 0.0013 | 0.6616 | 0.0062 | 0.0002 |
| Marital Status | <.0001 | 0.6129 | <.0001 | 0.0619 | <.0001 | 0.8649 | <.0001 | 0.7363 | <.0001 | 0.0040 |
| Histology | <.0001 | 0.3110 | 0.1697 | 0.0571 | <.0001 | 0.8234 | <.0001 | 0.3545 | 0.0040 | 0.0245 |
| Grade | <.0001 | 0.7976 | 0.3649 | 0.6966 | 0.0006 | 0.8537 | <.0001 | 0.6795 | 0.1901 | 0.2445 |
| ER Status | <.0001 | 0.5687 | 0.5450 | 0.4950 | <.0001 | 0.5008 | <.0001 | 0.4949 | 0.5847 | 0.5219 |
| PR status | <.0001 | 0.2181 | 0.4815 | 0.3948 | <.0001 | 0.1199 | <.0001 | 0.1707 | 0.4940 | 0.4224 |
| Laterality | 0.6450 | 0.9078 | 0.6530 | 0.4856 | 0.4829 | 0.5177 | 0.7670 | 0.4381 | 0.3947 | 0.6413 |
| Hypertension | 0.1851 | 0.4920 | 0.0012 | 0.6232 | 0.4696 | 0.9746 | 0.2898 | 0.9829 | 0.0024 | 0.2230 |
| MI | 0.0010 | 0.9088 | <.0001 | 0.0248 | <.0001 | 0.4962 | 0.0003 | 0.9286 | <.0001 | 0.1708 |
| Other IHD | 0.1830 | 0.8408 | 0.0022 | 0.0218 | 0.0104 | 0.5957 | 0.1133 | 0.6494 | 0.0205 | 0.0383 |
| Endo/Pericardium | 0.0373 | 0.7775 | <.0001 | 0.1214 | <.0001 | 0.8349 | 0.0395 | 0.9117 | <.0001 | 0.1177 |
| Cardiomyopathy | 0.1667 | 0.6704 | <.0001 | 0.4256 | 0.0023 | 0.4124 | 0.1651 | 0.8128 | 0.0004 | 0.4416 |
| ARR | 0.1500 | 0.6896 | <.0001 | 0.0028 | 0.0041 | 0.9195 | 0.1659 | 0.5790 | 0.0013 | 0.0152 |
| HF | <.0001 | 0.1389 | <.0001 | 0.6002 | <.0001 | 0.9027 | <.0001 | 0.2985 | <.0001 | 0.9952 |
| Stroke | 0.0765 | 0.1950 | <.0001 | 0.0903 | <.0001 | 0.6129 | 0.0676 | 0.1559 | <.0001 | 0.2995 |
| Stroke with Complications | 0.0004 | 0.5573 | <.0001 | 0.8050 | <.0001 | 0.9579 | 0.0004 | 0.4395 | <.0001 | 0.7934 |
| Atherosclerosis | 0.5877 | 0.8500 | 0.2030 | 0.9740 | 0.3520 | 0.9695 | 0.9579 | 0.7088 | 0.0955 | 0.7272 |
| Peripheral Vein | 0.1174 | 0.8155 | 0.0133 | 0.0714 | 0.0745 | 0.7736 | 0.2137 | 0.8243 | 0.1640 | 0.2486 |
| Aneurysm/Arterial Embolism/Thrombosis | 0.0047 | 0.1804 | <.0001 | 0.7952 | <.0001 | 0.4952 | 0.0084 | 0.2152 | <.0001 | 0.3422 |
| Non-Solid Caner | 0.9105 | 0.9005 | 0.4472 | 0.4690 | 0.6171 | 0.8267 | 0.6953 | 0.7548 | 0.7476 | 0.8502 |
| Pancreas Cancer | 0.0012 | 0.1801 | n/a | n/a | 0.4473 | 0.0319 | 0.1722 | 0.0949 | n/a | n/a |
| Kidney Cancer | 0.6390 | 0.2192 | 0.5139 | 0.4534 | 0.7795 | 0.1091 | 0.7470 | 0.2785 | 0.9869 | 0.1915 |
| Melanoma | 0.4679 | 0.9331 | n/a | n/a | 0.9819 | 0.6285 | 0.3823 | 0.9820 | n/a | n/a |

| | | | | | | | | | | |
|---|---|---|---|---|---|---|---|---|---|---|
| Lung Cancer | <.0001 | 0.6761 | <.0001 | 0.2649 | 0.0002 | 0.9342 | 0.0496 | 0.5605 | 0.0001 | 0.1634 |
| Colorectal Cancer | 0.1365 | 0.1945 | 0.0479 | 0.7586 | 0.0460 | 0.9754 | 0.1215 | 0.3331 | 0.1884 | 0.5113 |
| Other Solid Slow Progressive | 0.2463 | 0.7222 | 0.1080 | 0.8782 | 0.8236 | 0.8156 | 0.2817 | 0.6694 | 0.0335 | 0.5433 |
| Other Solid Fast Progressive | 0.1432 | 0.5604 | 0.8106 | 0.0009 | 0.6973 | 0.5759 | 0.9641 | 0.8273 | 0.5089 | 0.0286 |
| Secondary Malignant Neoplasm | 0.0005 | 0.7466 | 0.0520 | 0.9579 | 0.0955 | 0.2682 | 0.0790 | 0.7851 | 0.7186 | 0.9635 |
| Other Non-specified Cancer | 0.9667 | 0.6064 | 0.0936 | 0.7705 | 0.2110 | 0.9356 | 0.5887 | 0.4884 | 0.1343 | 0.6376 |
| COPD | 0.0102 | 0.5328 | 0.0007 | 0.9190 | 0.0006 | 0.4120 | 0.0148 | 0.9683 | 0.0085 | 0.6632 |
| Pulmonary Heart | 0.0632 | 0.8652 | <.0001 | 0.9894 | <.0001 | 0.9727 | 0.0686 | 0.7493 | <.0001 | 0.8892 |
| Pneumonia | 0.2592 | 0.5591 | <.0001 | 0.9384 | 0.0068 | 0.7993 | 0.6137 | 0.4130 | <.0001 | 0.6741 |
| Other Lung | 0.0031 | 0.4438 | <.0001 | 0.9234 | <.0001 | 0.0898 | 0.0289 | 0.5066 | <.0001 | 0.4685 |
| Dementia/Alzheimer | 0.0037 | 0.6106 | <.0001 | 0.0183 | <.0001 | 0.8404 | 0.0024 | 0.4036 | <.0001 | 0.2290 |
| Parkinson | 0.1731 | 0.5373 | 0.0034 | 0.7459 | 0.0096 | 0.4698 | 0.2162 | 0.5372 | 0.0032 | 0.0218 |
| Depression | 0.1231 | 0.2040 | 0.1867 | 0.4155 | 0.1461 | 0.2019 | 0.2497 | 0.1693 | 0.3601 | 0.3772 |
| Alcohol abuse | 0.0017 | 0.3792 | 0.4645 | 0.9983 | 0.0022 | 0.4224 | 0.0031 | 0.3014 | 0.2742 | 0.6468 |
| Drug/Medicine Abuse | 0.7925 | 0.0850 | 0.3883 | 0.4667 | 0.9541 | 0.0088 | 0.8888 | 0.0434 | 0.9103 | 0.9864 |
| Tobacco abuse | 0.5836 | 0.5384 | 0.8102 | 0.3402 | 0.8763 | 0.5675 | 0.8075 | 0.2681 | 0.9263 | 0.5285 |
| Diabetes | 0.3447 | 0.8990 | 0.0011 | 0.5123 | 0.0181 | 0.3961 | 0.2551 | 0.5989 | 0.0081 | 0.1172 |
| Electrolytes | <.0001 | 0.6125 | <.0001 | 0.4937 | <.0001 | 0.1795 | 0.0007 | 0.7529 | <.0001 | 0.7605 |
| Chronic Liver Disease | 0.3670 | 0.7484 | 0.2525 | 0.4555 | 0.1142 | 0.9901 | 0.3725 | 0.9034 | 0.1177 | 0.8624 |
| IBD | 0.0201 | 0.2762 | 0.5714 | 0.6743 | 0.1536 | 0.7144 | 0.0607 | 0.1853 | 0.7772 | 0.6945 |
| Ulcer | 0.5206 | 0.7523 | 0.4052 | 0.1976 | 0.2516 | 0.3331 | 0.5500 | 0.7058 | 0.2249 | 0.1961 |
| Gastric Bleeding | 0.1722 | 0.9699 | 0.3480 | 0.3047 | 0.1389 | 0.9932 | 0.1001 | 0.9811 | 0.8541 | 0.5230 |
| Renal Disease | 0.3413 | 0.9959 | <.0001 | 0.5004 | 0.0003 | 0.4870 | 0.8137 | 0.9931 | <.0001 | 0.4585 |
| Septicemia | <.0001 | 0.2422 | <.0001 | 0.0273 | <.0001 | 0.6753 | 0.0265 | 0.5062 | <.0001 | 0.0872 |
| HIV | 0.0033 | 0.1552 | <.0001 | 0.6059 | <.0001 | 0.7143 | 0.0026 | 0.1585 | <.0001 | 0.4578 |
| Anemia | 0.0770 | 0.4891 | <.0001 | 0.8411 | 0.0013 | 0.5173 | 0.2194 | 0.2998 | <.0001 | 0.3659 |
| Upper/Lower Limb Fracture | 0.8160 | 0.6516 | 0.4368 | 0.6038 | 0.6277 | 0.2012 | 0.9758 | 0.5964 | 0.3874 | 0.5810 |
| RA | 0.7993 | 0.4248 | 0.9102 | 0.9491 | 0.7327 | 0.1333 | 0.8058 | 0.1885 | 0.7998 | 0.7212 |
| Senility | 0.3665 | 0.6777 | 0.1877 | 0.2067 | 0.6637 | 0.6922 | 0.7916 | 0.3522 | 0.6879 | 0.0811 |
| Low Weight | 0.0385 | 0.5720 | <.0001 | 0.2737 | <.0001 | 0.2542 | 0.1533 | 0.4499 | <.0001 | 0.2613 |
| Obesity | 0.6956 | 0.4617 | 0.1070 | 0.6773 | 0.5103 | 0.6963 | 0.9937 | 0.4588 | 0.2110 | 0.2025 |
| Lower-poverty-level | 0.7580 | 0.4918 | 0.6397 | 0.0603 | 0.7729 | 0.5574 | 0.7925 | 0.5199 | 0.8930 | 0.1139 |
| Higher Income | 0.4868 | 0.6681 | 0.2693 | 0.4671 | 0.2966 | 0.9129 | 0.7533 | 0.5813 | 0.1671 | 0.2521 |
| Lower-fraction-of-black Population | 0.0952 | 0.3375 | <.0001 | 0.7185 | 0.0018 | 0.3858 | 0.1407 | 0.2367 | <.0001 | 0.0165 |
| More Educated | 0.5654 | 0.9766 | 0.7721 | 0.3786 | 0.4370 | 0.9233 | 0.4961 | 0.9680 | 0.8275 | 0.2388 |

**Supplementary Table 5**. Sensitivity Studies: estimates of ORs for alternative methods or patient subgroups (pseudorandomized cohorts).

| | Group AS1 | | Group AS2 | |
|---|---|---|---|---|
| | OS | BCS | OS | BCS |
| Main model (conside with Table 2) | 0.99 ( 0.93, 1.05) | 0.81 ( 0.62, 1.05) | 3.54 ( 3.29, 3.82) | 10.7 ( 8.63, 13.4) |
| Age group is additional covariate | 1.09 ( 1.03, 1.16) | 0.89 ( 0.69, 1.16) | 2.81 ( 2.60, 3.02) | 8.91 ( 7.12, 11.1) |
| Age-follow-up | 1.09 ( 1.03, 1.16) | 0.89 ( 0.68, 1.16) | 2.66 ( 2.47, 2.86) | 8.17 ( 6.53, 10.2) |
| Age-follow-up with age group as covariate | 1.08 ( 1.01, 1.14) | 0.88 ( 0.68, 1.15) | 2.69 ( 2.50, 2.90) | 8.38 ( 6.68,10.5) |
| Alternative definition of DCIS | 1.37 ( 1.30, 1.45) | 1.24 ( 0.98, 1.58) | 4.53 ( 4.24, 4.84) | 10.3 ( 8.30,12.8) |
| Medicare death | 0.95 ( 0.89, 1.01) | 0.81 ( 0.62, 1.05) | 3.56 ( 3.31, 3.82) | 10.7 ( 8.63, 13.4) |
| Subroup: Metropolian | 0.99 ( 0.93, 1.06) | 0.90 ( 0.68, 1.18) | 3.55 ( 3.29, 3.83) | 10.9 ( 8.63, 13.6) |
| Subroup: Non-Metropolian | 1.00 ( 0.86, 1.16) | 0.40 ( 0.18, 0.93) | 2.54 ( 1.69, 3.80) | 5.63 ( 1.86, 17.1) |
| Subroup: Age 65-69 | 1.20 ( 1.03, 1.40) | 0.00 ( 0.00, . ) | 4.89 ( 3.93, 6.09) | 8.76 ( 4.25, 18.0) |
| Subroup: Age 70-74 | 0.67 ( 0.58, 0.77) | 1.35 ( 0.86, 2.11) | 3.47 ( 2.95, 4.10) | 21.4 ( 14.6, 31.5) |
| Subroup: Age 75-79 | 1.21 ( 1.08, 1.35) | 0.95 ( 0.53, 1.68) | 3.61 ( 3.14, 4.15) | 9.11 ( 5.64,14.7) |
| Subroup: Age 80-84 | 1.08 ( 0.95, 1.22) | 0.76 ( 0.42, 1.37) | 2.61 ( 2.22, 3.07) | 1.83 ( 0.86, 3.91) |
| Subroup: Age 85+ | 1.49 ( 1.28, 1.73) | 1.51 ( 0.84, 2.71) | 1.64 ( 1.39, 1.94) | 5.03 ( 3.08, 8.20) |
| Subroup: Midwest | 0.85 ( 0.74, 0.98) | 0.94 ( 0.54, 1.64) | 3.96 ( 3.29, 4.75) | 13.7 ( 8.23, 22.7) |
| Subroup: Northeast | 0.75 ( 0.65, 0.87) | 0.95 ( 0.57, 1.57) | 3.12 ( 2.53, 3.85) | 5.65 ( 3.04, 10.5) |
| Subroup: South | 0.93 ( 0.81, 1.06) | 0.78 ( 0.40, 1.52) | 2.27 ( 1.96, 2.62) | 19.4 (12.6, 29.8) |
| Subroup: West | 1.30 ( 1.19, 1.43) | 0.57 ( 0.36, 0.92) | 4.89 ( 4.38, 5.46) | 5.22 ( 3.41, 7.98) |
| Subroup: Grade Moderately differentiated | 1.36 ( 1.21, 1.52) | 2.23 ( 1.38, 3.62) | 4.28 ( 3.73, 4.90) | 13.2 ( 8.29, 21.0) |
| Subroup: Grade Poorly differentiated | 1.27 ( 1.11, 1.46) | 0.38 ( 0.17, 0.84) | 3.80 ( 3.29, 4.38) | 18.9 ( 12.5, 28.7) |
| Subroup: Histology 8500 | 1.10 ( 1.02, 1.18) | 1.18 ( 0.87, 1.61) | 3.34 ( 3.02, 3.68) | 13.7 ( 10.4, 18.1) |
| Subroup: Histology 8501 | 1.12 ( 0.97, 1.30) | 0.11 ( 0.03, 0.42) | 4.94 ( 4.29, 5.69) | 7.29 ( 4.73, 11.2) |
| Subroup: Married | 1.16 ( 1.06, 1.28) | 0.71 ( 0.46, 1.11) | 5.67 ( 4.99, 6.44) | 21.3 ( 15.0, 30.2) |
| Subroup: non-Married | 0.93 ( 0.86, 1.01) | 0.92 ( 0.67, 1.28) | 2.62 ( 2.39, 2.86) | 6.37 ( 4.80, 8.44) |
| Subroup: no highly-lethal cancer at baseline* | 0.88 ( 0.82, 0.94) | 0.58 ( 0.41, 0.81) | 3.65 ( 3.37, 3.96) | 12.0 ( 9.39, 15.4) |
| Subroup: no CVD at baseline* | 0.97 ( 0.88, 1.07) | 0.52 ( 0.33, 0.83) | 4.97 ( 4.41, 5.60) | 19.1 (13.8, 26.2) |
| Subroup: no CRVD at baseline* | 0.86 ( 0.79, 0.93) | 0.56 ( 0.39, 0.79) | 3.52 ( 3.18, 3.89) | 6.04 ( 4.34, 8.43) |
| Subroup: no lung diseases at baseline* | 0.88 ( 0.80, 0.98) | 0.64 ( 0.42, 0.99) | 2.05 ( 1.76, 2.39) | 2.87 ( 1.71, 4.81) |
| Subroup: no NDD at baseline* | 1.08 ( 1.02, 1.16) | 0.89 ( 0.67, 1.17) | 3.40 ( 3.12, 3.71) | 6.39 ( 4.81, 8.49) |
| Subroup: no Diabetes at baseline | 0.94 ( 0.87, 1.02) | 0.70 ( 0.50, 0.98) | 3.54 ( 3.21, 3.89) | 7.82 ( 5.79,10.6) |
| Subroup: no Renal disease at baseline | 1.02 ( 0.96, 1.10) | 0.42 ( 0.29, 0.60) | 2.88 ( 2.62, 3.17) | 12.3 ( 9.65, 15.6) |

* Excluded cancer were Non-Solid, Pancreas, Kidney, Lung, Colorectal cancers, Melanoma and Secondary malignant neoplasm; excluded CVD were MI Endo/Pericardium Cardiomyopathy HF and Aneurysm/ Embolism/Thrombosis; excluded CRVD were Stroke and Stroke with complications; excluded lung diseases were COPD Pulmonary Heart Pneumonia Other Lung diseases; and excluded NDD were Dementia/Alzheimer's and Parkinson's diseases.

**Supplementary Table 6.** Codes to identify breast cancer treatment in Medicare data

| Variable | ICD-9-Diagnosis | ICD-9-Procedure | CPT/HCPCS |
|---|---|---|---|
| Mastectomy | | 85.23, 85.41-85.48 | 19180, 19182, 19200, 19220, 19240, 19271, 19272, 19300, 19303- 19307 |
| Lumpectomy | | 85.12, 85.2, 85.21, | 19101, 19110, 19120, 19125, 19126, 19160, 19162, |

| | | | |
|---|---|---|---|
| | | 85.22, 85.24, 85.25 | 19301, 19302 |
| Radiation therapy | V58.0, V66.1, V67.1 | 92.2, 2.20–92.27, 92.29, 92.3, 92.30–92.39, 92.4, 92.41 | G0174, G0251, G0339, G0340<br>77371–77373, 77401–77499, 77750–77799 |

**Supplementary Table 7:** ICD-9-CM codes for comorbid diseases

| System | Specific disease and ICD-9-CM |
|---|---|
| **Circulatory** | Hypertension (401-405), Myocardial infarction (MI) (410), Other ischemic heart diseases (IHD) (411, 413, 414), Endo/Pericardium (420-424), Cardiomyopathy (425, 429, 422), Cardiac Arrhythmias (ARR) (426, 427), Heart Failure (428), Stroke (430-437, 348), Stroke with complications (438, 342, 344), Atherosclerosis (440, 272), Peripheral Vein (451-454, 456, 459), Aneurysm/Arterial Embolism/Thrombosis (441-445, 447, 557). |
| **Neoplasms** | Non-Solid (200-208), Pancreas (157), Kidney (189), Melanoma (172), Lung (162), Colorectal (153, 154), Other solid fast progressive (150-152, 155, 156, 158, 159, 163-165, 170, 171, 175, 176, 179, 182, 183, 188, 191, 192, 194, 195, 209.0-209.3), Other solid slow progressive (140-149, 160, 161, 173, 180, 184, 186, 187, 190, 193), Secondary malignant neoplasm (196-198), Other Non-specified (199, 238, 239). |
| **Respiratory** | Chronic obstructive pulmonary disease (COPD) (490-496), Pulmonary Heart (415, 416, 514), Pneumonia (480-488), Other Lung (510-519). |
| **Mental** | Dementia/Alzheimer (290,294,331.0,331.1,331.2,331.9), Parkinson (332, 331.82), Depression (301.12, 300.4,309.0,309.1,296.2,296.3,296.5,298.0,311) Alcohol abuse (305.0, 357.5, 425.5, 535.3, 571.0-571.3, 291, 303, 980), Drug/Medicine Abuse (292, 304.1, 305.4, 305.8, 305.9), Tobacco abuse(989.84, V15.82, 305.1). |
| **Endocrine** | Diabetes (250), Electrolytes (276). |
| **Digestive/** | Chronic Liver (571-573, 0702-0707, 0709), Inflammatory Bowel Disease (IBD) (556, 555, 558, 538), Ulcer (531-534), |
| **Renal** | Gastrointestinal hemorrhage/bleeding (578), Renal (580-589, V56, 593). |
| **Infectious** | Septicemia (038), HIV (042). |
| **Blood** | Anemia (286.5, 286.7, 286.9, 280, 281, 283-285, 287, 289, V78). |
| **Injury** | Upper/Lower Limb Fracture (V82.81, V54.13, V13.5, 905.2-905.5, 810-813, 817-821, 823-825, 827, 828, 733, V54). |
| **Other** | Rheumatoid arthritis (RA) (714,725), Senility (797), Low Weight (783.2, 783.7, 799.4, 783.0, 783.3, 783.9, 260-263), Obesity (278). |
