# Supplementary material for: A medicare-based comparative mortality analysis of active surveillance in older women with DCIS
Source: NPJ Breast Cancer. 2020 Oct 30;6:57. doi: 10.1038/s41523-020-00199-0 (PMC7599206; doi:10.1038/s41523-020-00199-0)
Supplement: Supplementary file 2 — Reporting Summary [file 41523_2020_199_MOESM2_ESM.pdf]

nature research

Corresponding author(s): Igor Akushevich

Last updated by author(s): May 17, 2020

Reporting Summary

Nature Research wishes to improve the reproducibility of the work that we publish. This form provides structure for consistency and transparency in reporting. For further information on Nature Research policies, see our Editorial Policies and the Editorial Policy Checklist.

Statistics

For all statistical analyses, confirm that the following items are present in the figure legend, table legend, main text, or Methods section.

n/a	Confirmed	
☐	☒	The exact sample size (*n*) for each experimental group/condition, given as a discrete number and unit of measurement
☐	☒	A statement on whether measurements were taken from distinct samples or whether the same sample was measured repeatedly
☐	☒	The statistical test(s) used AND whether they are one- or two-sided<br>*Only common tests should be described solely by name; describe more complex techniques in the Methods section.*
☐	☒	A description of all covariates tested
☐	☒	A description of any assumptions or corrections, such as tests of normality and adjustment for multiple comparisons
☐	☒	A full description of the statistical parameters including central tendency (e.g. means) or other basic estimates (e.g. regression coefficient) AND variation (e.g. standard deviation) or associated estimates of uncertainty (e.g. confidence intervals)
☐	☒	For null hypothesis testing, the test statistic (e.g. *F*, *t*, *r*) with confidence intervals, effect sizes, degrees of freedom and *P* value noted<br>*Give P values as exact values whenever suitable.*
☒	☐	For Bayesian analysis, information on the choice of priors and Markov chain Monte Carlo settings
☒	☐	For hierarchical and complex designs, identification of the appropriate level for tests and full reporting of outcomes
☒	☐	Estimates of effect sizes (e.g. Cohen's *d*, Pearson's *r*), indicating how they were calculated

Our web collection on statistics for biologists contains articles on many of the points above.

Software and code

Policy information about availability of computer code

Data collection	Analysis and data management was done using SAS software version 9.4. Data was drawn from secondary sources.
Data analysis	Analysis and data management was done using SAS software version 9.4.

For manuscripts utilizing custom algorithms or software that are central to the research but not yet described in published literature, software must be made available to editors and reviewers. We strongly encourage code deposition in a community repository (e.g. GitHub). See the Nature Research guidelines for submitting code & software for further information.

Data

Policy information about availability of data

All manuscripts must include a data availability statement. This statement should provide the following information, where applicable:

- Accession codes, unique identifiers, or web links for publicly available datasets
- A list of figures that have associated raw data
- A description of any restrictions on data availability

The manuscript is based on Medicare-linked SEER data. The Centers for Medicare and Medicaid Services do not allow the redistribution of their data by researchers. However, upon request and after confirmation that the requestor has the appropriate DUAs in place, as well as IRB approval at his/her institution the authors will provide SAS code that will allow the re-generation of the data in a form appropriate to the replication of the current analysis.

Field-specific reporting

Please select the one below that is the best fit for your research. If you are not sure, read the appropriate sections before making your selection.

☒ Life sciences ☐ Behavioural & social sciences ☐ Ecological, evolutionary & environmental sciences

For a reference copy of the document with all sections, see nature.com/documents/nr-reporting-summary-flat.pdf

Life sciences study design

All studies must disclose on these points even when the disclosure is negative.

Sample size	Detailed description of sample sizes of total sample and comparison groups are given in Methods Section and in Figure 1.
Data exclusions	Patient selection procedure was described in details in Methods Section as well as in Supplementary Figure S1.
Replication	N.A. The study involves secondary data analysis and no other datasets of comparable detail and power exist for this population.
Randomization	The propensity score-based pseudorandomization was used. The approach is described in detail in Methods Section
Blinding	The authors were blinded to the group of researchers collected data.

Reporting for specific materials, systems and methods

We require information from authors about some types of materials, experimental systems and methods used in many studies. Here, indicate whether each material, system or method listed is relevant to your study. If you are not sure if a list item applies to your research, read the appropriate section before selecting a response.

Materials & experimental systems

n/a	Involved in the study	
☒	☐	Antibodies
☒	☐	Eukaryotic cell lines
☒	☐	Palaeontology and archaeology
☒	☐	Animals and other organisms
☐	☒	Human research participants
☒	☐	Clinical data
☒	☐	Dual use research of concern

Methods

n/a	Involved in the study	
☒	☐	ChIP-seq
☒	☐	Flow cytometry
☒	☐	MRI-based neuroimaging

Human research participants

Policy information about studies involving human research participants

Population characteristics	We analyzed health effects of female patients diagnosed with breast cancer at age 65 or older
Recruitment	Required population approximately represents female population of older adults. Details are given in Methods section
Ethics oversight	Duke University IRB approved study protocol

Note that full information on the approval of the study protocol must also be provided in the manuscript.
